# A pilot randomized controlled trial comparing bismuth iodine paraffin paste external ear pack and no ear pack after middle ear surgery

**DOI:** 10.1007/s00405-013-2812-6

**Published:** 2013-12-14

**Authors:** Faisal Javed, Russell Whitwell, Daniel Hajioff, Philip Robinson, David Rea, Iain Macleod, Paul White, Desmond A. Nunez

**Affiliations:** 1 Department of Ear, Nose and Throat, Royal United Hospital, Combe Park, Bath, BA1 3NG UK; 2 Department of ENT, Southmead Hospital, Bristol, BS10 5NB UK; 3 Applied Statistics Group, University of West of England, Bristol, BS16 1QY UK; 4 Division of Otolaryngology-Head and Neck Surgery, University of British Columbia, Vancouver, Canada; 5 ENT Department, North Bristol Hospitals, NHS Trust, Bristol, UK

**Keywords:** Ear packing, BIPP, Randomized controlled trial, Canal wall-up ear surgery, Visual analogue scores, Ear blockage

## Abstract

To pilot a substantive randomized control trial comparing post-operative external ear canal pack with no ear pack after middle ear surgery, 32 adults undergoing primary posterior bony canal wall preserving middle ear surgery were randomized to have either a bismuth iodoform paraffin paste pack or no ear pack post-operatively. Outcome measures included clinician- and patient-recorded visual analogue scale scores for ear signs and symptoms at 3 weeks and 3 months and audiometric findings at 3 months post-operatively. There was no statistically significant inter-group difference in 3-week clinician and patient cumulative scores for ear signs and symptoms. There was also no significant difference in graft take rate, appearance of ear canals and audiometric results in either group at 3 months. No difference in ear symptoms, clinician findings or hearing was demonstrated between patients with a post-operative pack compared to those without a pack following middle ear surgery in this pilot study.

## Introduction

Packing of the external auditory canal (EAC) after major ear surgery is an established practice in otological surgery. The types of packing vary, with a wide range of individual preferences based more on tradition than evidence [1]. It is believed that packing of the EAC has several functions such as: adaptation and fixing the canal skin flap and skin grafts, prevention of adhesions, granulations, blunting or lateralization of grafts and maintaining the shape and volume of the fibro-cartilaginous part of the ear canal [2]. Conversely, adverse reactions to ear packing have also been reported, namely patient discomfort, infection, hypersensitivity reactions and deformity [3–5]. Moreover, patients having ear packing need a follow-up outpatient visit after surgery for removal of non-absorbable packs.

A study performed in children suggests that packing after ear surgery may be safely abandoned [1]. In our practice we have seen many patients (particularly children) who remove the pack themselves almost immediately after surgery presumably due to discomfort with no adverse outcome noticed.

This raises the question of whether ear packing after middle ear surgery is beneficial. We performed a pilot study into the difference in recorded symptoms, clinical findings and audiometric outcomes between patients who have and do not have ear packing after major ear surgery as there were no comparable studies on which power calculations could be based.

## Materials and methods

### Ethical considerations

Patients who gave informed written consent were recruited after obtaining the approval of the Southmead Hospital Research Ethics Committee. Ethical committee approval was given to recruit a total of 32 patients in this pilot study to provide data for a power calculation.

### Inclusion criteria and exclusion criteria

Adults undergoing primary middle ear surgery with preservation of the posterior bony canal wall ± closed cavity mastoidectomy were included. This specifically included patients undergoing myringoplasty, ossiculoplasty, stapedectomy or canal wall-up (combined approach) mastoidectomy. Surgical approaches included permeatal, endaural and postaural.

Exclusion criteria included patients with grossly abnormal ear canal anatomy, history of otological malignancy, immunosuppression and those unable or unwilling to return for follow-up. Patients undergoing meatoplasty, canalplasty or operations where no packing is currently used, e.g. grommets, were also excluded.

### Randomization

A column of numbers in the uniform random numbers table generated by the Numerical Algorithm Group routines quoted in Machin and Campbell’s Statistical *Tables for the Design of clinical trials* was used in sequence to allocate consecutive trial recruits either to no pack when the column number was odd or pack when the column number was even for a total of 32 potential trial applicants [6].

### Surgical technique

A layer of absorbable gelatine sponge (Spongostan) was placed over the tympanic membrane and tympanomeatal flap, once these were positioned at the end of the surgery. The surgeon was then informed of the study decision whether to pack or not. The allocation was revealed by telephoning the study coordinator. Patients randomized to having ear packs had a 4 in. length of 1.25 cm wide ribbon gauze impregnated with bismuth iodoform paraffin paste (BIPP) inserted into the operated external ear canal at the end of the surgery. BIPP was chosen, as it is still one of the most widely used ear packs after middle ear surgery in the UK [3]. In the other group, no ear pack was inserted. Ear packs were removed 3 weeks after surgery.

### Outcome measures

Three weeks following surgery, patients were asked to complete visual analogue scales (VAS) quantifying each of the three main symptoms: pain, discharge and itching (see “Appendices 1, 2”). The ‘ear pack’ group patients were also asked to quantify the pain on pack removal. The clinician reviewing the patient completed the visual analogue scales, which quantified each of the three main signs: discharge, granulation tissue and erythema (see “Appendices 1, 2”). Clinicians also recorded their findings about the integrity and position (lateralized or not) of the tympanic membrane and the shape of the EAC (deformed or not) along with any evidence of meatal stenosis. Meatal stenosis was defined as a significant narrowing of external ear canal opening post-operatively impairing self-cleaning of EAC.

All patients were further reviewed at 3 months when the patients and clinicians again recorded visual analogue scores as above.

### Audiometric assessment

All patients had audiometric evaluation pre-operatively and 3 months post-operatively. This included air-conduction (AC) thresholds and bone-conduction (BC) thresholds with masking according to the British Society of Audiology guidelines [7]. AC and BC obtained at the same time were used for calculating the air–bone gap (ABG). AC and BC were checked at 0.5, 1, 2 and 3 kHz according to the American Academy of Otolaryngology-Head and Neck Surgery guidelines [8]. When thresholds at 3 kHz were not available, they were replaced with 4 kHz. The post-operative audiograms at 3 months were compared with pre-operative audiograms obtained within 3 months of the patient’s surgery.

### Statistical analysis

All data were analysed by SPSS Version 19.02. Inter-group comparisons of 3 week and 3 month post-op clinician and patient VAS scores were performed using independent sample *t* tests. Fisher’s exact test was used to compare categorical data, namely integrity and lateralization of tympanic membrane, presence of meatal stenosis, infection and deformity of the ear canal.

Inter-group comparisons of (pre–post treatment) mean change in four frequencies (0.5, 1, 2 and 3 or 4 kHz), average air-conduction thresholds, bone-conduction thresholds and air–bone gap were assessed by the independent sample Student’s *t* test.

Analysis was performed using the intention-to-treat principle (ITT), sorting all subjects by their original randomization group irrespective of the treatment type actually used.

## Results and analysis

**Fig. 1:**
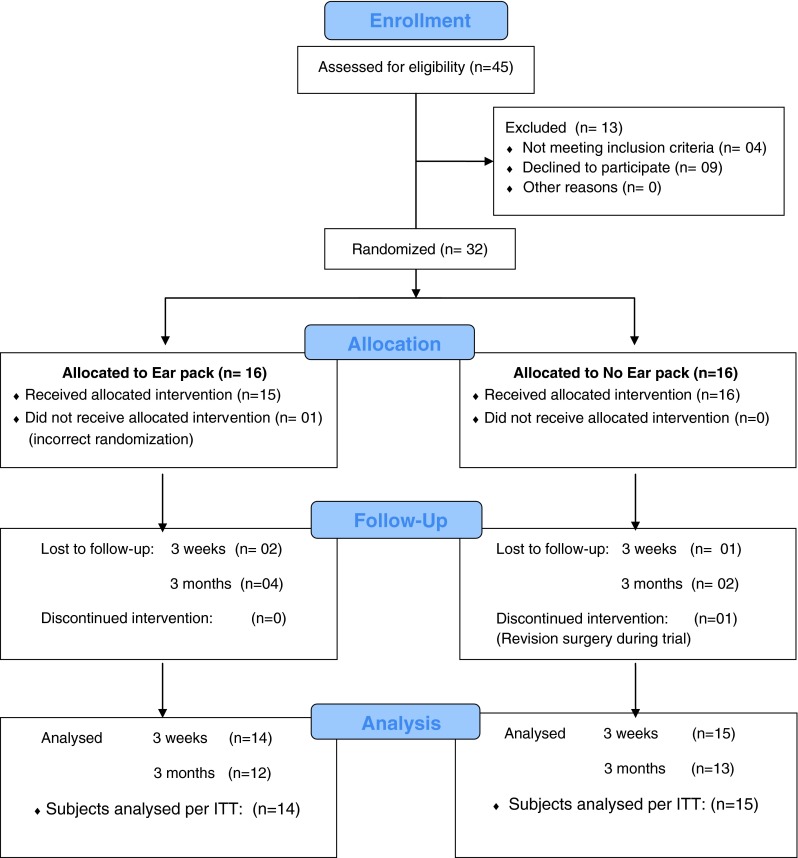
Consort flow diagram

Thirty-two consenting patients (15 male and 17 female; mean age 47 years: range 21–75) who were scheduled for major ear surgery were prospectively recruited to the study (Fig. 1). No adverse event or harm occurred to the patients during this trial.

Sixteen patients were randomized to ear packing (7 male and 9 female, age range 35–67) and 16 to the ‘no ear packing’ group (8 male, 8 female, age range 21–75). One patient had revision surgery within 3 months of recruitment. The revision surgery was not related to the trial. In one patient the randomization allocation decision was incorrectly conveyed to the surgeon. These two patients were included in the intention-to-treat (ITT) analysis.

The treatment groups were demographically similar: mean age of 45 and 48 years in the ‘ear pack’ and ‘no ear pack’ groups, respectively, with no statistically significant difference in the distribution of patients by sex. A list of the different surgical procedures and surgical approaches utilized in each group is shown in Tables 1 and 2.

**Table 1:** Summary of procedures performed in both groups

Procedures	Ear packing *n* = 16	No ear packing *n* = 16	*P* value
Combined approach tympanoplasty	5	6	0.7
Myringoplasty	7	5	0.7
Stapedotomy	3	4	1.0
Osciculoplasty	1	1	1.0

**Table 2:** Summary of different surgical approaches in both groups

Surgical approach	Ear packing *n* = 16	No ear packing *n* = 16	*P* value
Postaural	8	8	0.7
Endaural	5	6	1.0
Permeatal	3	2	0.6

At 3 weeks, VAS on 14 patients from the ‘ear pack’ and 15 patients from the ‘no ear pack’ group were available for analysis. At 3 months, VAS on 12 patients and 13 patients randomized to ‘ear pack’ and ‘no ear pack’, respectively, were analysed. Audiometric data were available for 25 patients at 3 months: 11 patients from the ‘ear pack’ group and 14 patients from the ‘no ear pack’ group.

**Fig. 2:**
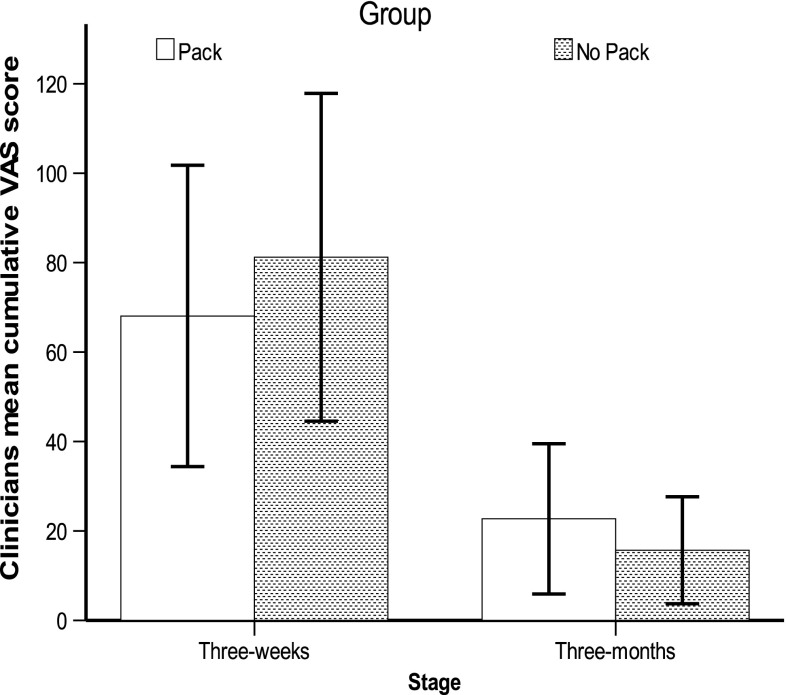
Summary of clinician mean VAS score (*error bars*; confidence intervals)

There was no statistically significant inter-group difference in 3-week clinicians’ mean cumulative VAS on ITT analysis: 64 mm for ear packing group versus 81 mm for no ear packing group (*p* = 0.5) (Fig. 2). Analysis of the 3 month clinicians’ mean cumulative VAS did not show a statistically significant difference between both groups (22.7 mm for ear packing group vs 17 mm for no ear packing group, *p* = 0.4) (Fig. 2).

**Fig. 3:**
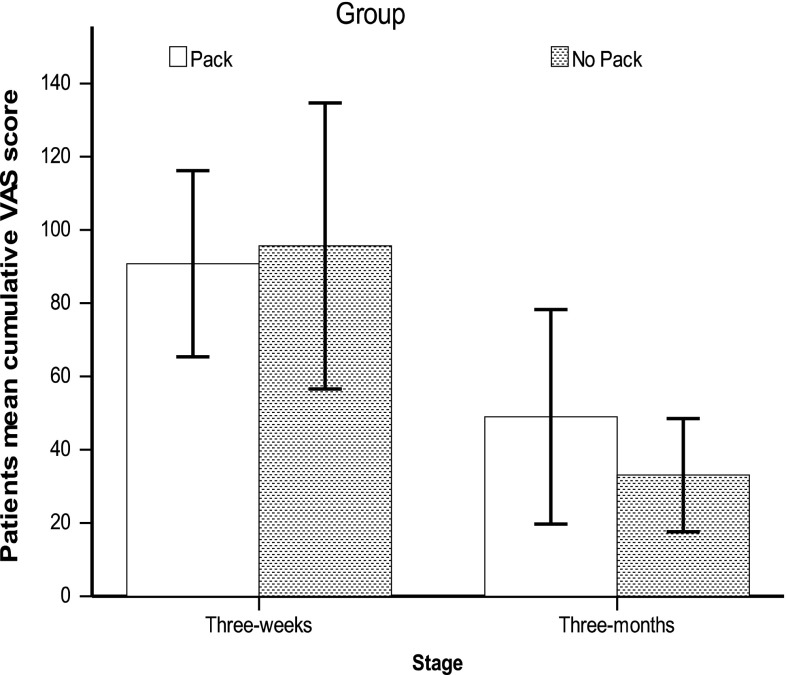
Summary of patients’ mean VAS score (*error bars*; confidence intervals)

Analysis of patients’ VAS at 3 weeks showed no significant difference in mean cumulative VAS scores between both groups on ITT analysis: 95 mm for ear pack group and 98 mm for no ear pack group (*p* = 0.6) (Fig. 3). Two patients from the ‘no ear pack’ group presented of their own accord with ear infection within the first 3 weeks of surgery. Both of these patients were treated successfully with topical antibiotic drops. This outcome was not statistically different from the ear pack group (*p* = 0.4). In the ‘ear pack’ group patients, the mean pain score on pack removal was 26 mm (out of 100 mm). There is of course no equivalent measure in the control group with which to compare this outcome. The mean 3 month patients mean cumulative VAS scores for all three outcome measures also did not differ significantly (49 mm for ‘ear pack’ group vs 35.5 mm for ‘no ear pack’ group, *p* = 0.4).

There was no significant difference in ear canal deformity and meatal stenosis between both groups at 3 months (Table 3). One patient in the ‘ear pack’ group was recorded to have meatal narrowing at 3 months, but with no adverse consequence. One patient in the ‘no ear pack’ group was recorded to have a deformed ear canal at 3 months. No adverse consequence was apparent in this case either. Unsuccessful graft take was reported in one patient in the ‘ear pack’ group and two patients in the ‘no pack’ group at 3 months (*p* = 1.0).

**Table 3:** Summary of secondary outcome measures

Outcome measure	Ear packing group *n* = 12	No ear pack group *n* = 13	*P* value
Graft failure	1	2	1.0
Lateralized tympanic membrane	0	0	1.0
Deformed ear canal/external meatal stenosis or narrowing	1	1	1.0

The pure tone audiometric data did not show statistically significant differences between groups (findings summarized in Table 4). The mean post-operative air–bone gap was 13.0 db HL (SD 6.29, 95 % CI 8.77–17.23) in the ear packing group compared to 19.1 db HL (SD 14.97, 95 % CI 10.52–27.8) in the ‘no ear packing’ group (*p* = 0.2). Similarly, all other comparisons (change in ABG, change of air-conduction thresholds, change of bone-conduction thresholds) did not show statistically significant difference between the groups.

**Table 4:** Summary analysis of audiometric outcomes

Audiometric outcome	Ear pack group *n* = 11	No ear pack group *n* = 14	*P* value
Mean pre-operative ABG	22.60 db (SD 7.95) 95 % CI = 17.26–27.94	23.12 db (SD 14.83) 95 % CI = 14.56–31.68	0.9
Mean post-operative ABG	13.0 db (SD 6.29) 95 % CI = 8.77–17.23	19.16 db (SD 14.97) 95 % CI = 10.52–27.8	0.2
Mean ABG change (pre-operative ABG–post-operative ABG)	9.6 db (SD 12.45) 95 % CI = 1.23–17.96	3.96 db (SD 11.95) 95 % CI = −2.9 to 10.9	0.2
Mean AC change	11.4 db (SD 10.93) 95 % CI = 4.05–18.74	3.5 db (SD 12.05) 95 % CI = −3.45 to 10.46	0.1
Mean BC change	1.8 db (SD 6.74) 95 % CI = −2.72 to 6.39	−0.5 db (SD 5.63) 95 % CI = −3.75 to 2.75	0.6

*ABG* air–bone gap, *AC* air conduction, *BC* bone conduction

## Discussion

Ear packing after middle ear surgery is a common practice. Though ear packing has been suggested to help avoid ear canal deformity or external ear canal stenosis post-operatively by stabilizing the grafts and skin flaps, one might argue that there is also a risk of inadvertently disturbing the graft during the packing process with a subsequent chance of graft displacement during pack removal. In our pilot study, two patients in the no ear pack group had persistent perforations after surgery. One patient had a subtotal perforation, which was closed using tragal cartilage with perichondrium. The other patient had a butterfly inlay cartilage graft for a small central perforation. The size of the perforation has been reported as an important factor influencing the success rate of the procedure and is better in patients with smaller perforations than those with large perforations [9]. The success rate for butterfly graft myringoplasty is also variable and lies between 43 and 100 % [10]. Thus, one might argue that the persistent perforation seen in these two patients might be related to the size of the perforation and technique used for closure rather than the absence of a BIPP pack. The patient in the ear pack group who had a persistent perforation post-operatively underwent the procedure using temporalis fascia graft with underlay technique.

The three most commonly used surgical approaches to the middle ear and mastoid are: transcanal, endaural and postauricular [11]. Soft tissue dissection in and around the external ear canal opening is an important surgical step to achieve access with both endaural and postaural approaches, thus raising the possibility of an adverse outcome like narrowing or stenosis if no post-operative ear pack is used. In our pilot study, patients in both groups were operated through permeatal, endaural or postaural approaches with no significant difference seen in either post-operative ear canal deformity or meatal stenosis between both groups. Hiroven et al.’s [12] study of patients undergoing stapes surgery via a permeatal approach also found no significant disadvantage to not packing the ears.

There are various types of ear packs used in middle ear surgery. Non-absorbable packs include pope wick, silastic sheet, ribbon guaze mixed with antibiotic and/or antiseptic ointments or creams and the time-honoured BIPP packs. Absorbable packs can be in the form of either gelatin sponge (Spongostan) or Tri-Adcortyl/Polyfax ointment. Non-absorbable packs are usually removed in the first 2–3 weeks after surgery. This is usually done in outpatient clinics without any anaesthetic. The procedure can be uncomfortable. Other risks include bleeding and displacement of grafts if it is tethered to the pack. In a study by Zeitoun et al. [13], BIPP packs were found to be very uncomfortable for patients post-operatively. They were also fairly painful to remove. In our small study there was an expression of pain measured on the VAS score during pack removal, however as there was no control, the significance of this cannot be ascertained from our trial.

Borgstein et al.’s [1] retrospective paediatric study of 107 patients did not report any significant adverse findings related to not packing the ears. They had an infection rate of 7.5 %, all of whom were managed successfully with topical antibiotics. In our pilot study, two patients from the no ear pack group presented with ear infection before their intended 3 weeks outpatient appointment. Both were managed successfully with topical eardrops and did not have any adverse outcome recorded at their 3 month appointments. One patient in the ear pack group also had a high clinician VAS (cumulative VAS score: 238 mm) recorded after his pack removal at 3 weeks indicating that there was an external ear canal infection, but as his ear canal was occluded with an ear pack, no external ear discharge was noted by the patient. Therefore using ear packing does not abolish the risk of a post-operative ear infection.

We used cumulative VAS in our pilot study as this has been shown to be reliable in determining the extent of post-operative symptoms. This has been used successfully by one of the authors to assess ear blockage, pain, itch and discharge in patients with otitis externa [14].

When packing is placed in the EAC, a unilateral hearing loss due to blockage of sound transmission and loss of external ear resonance is expected. For most patients, this is usually not concerning as it is accepted as a temporary effect, but when the operated ear is the only hearing ear or better hearing ear, this might be a more significant problem [15]. Whilst a degree of hearing impairment is likely to occur as a result of blood clots and exudates, this is likely to be exacerbated by placing packing in the ear canal. Cho et al. [15] in their study on the effect of ear packing on hearing have reported a significant increase in AC and ABG (>40 db) with ear packs in situ. We did not study the effect of the ear pack whilst in situ on hearing.

### Weakness of the study

As this is a small pilot study of 32 patients, there is a risk of type ΙΙ error, i.e. failure to demonstrate a difference in the outcome measures between both groups when such a difference may actually exist.

### Power calculation

Analysis of quantitative variables, i.e. clinician VAS scores at 3 months using the independent samples *t* test (assuming equal variances) shows that the observed differences in mean changes is not significant. However, the effect size is estimated to be approximately 0.6 (i.e. Cohen’s *d* approximately 0.6 which indicates a moderate effect size). If this estimated effect is a good estimate and a follow-on study was to be conducted with equal allocation ratio between the two arms, then complete data on *n* = 60 for each arm would be needed to obtain 90 % power.

Analysis of patients VAS scores at 3 months using the independent samples *t* test (assuming equal variances) shows that the observed differences in mean changes is also not significant. However, the effect size is estimated to be approximately 0.6 (i.e. Cohen’s *d* approximately 0.6 which indicates a moderate effect size). For this parameter, the same sample size outlined above will be required to achieve 90 % power.

## Conclusion

No difference in ear symptoms, clinician findings or hearing was demonstrated between patients with a post-operative pack compared to those without a pack following middle ear surgery in this pilot study. This pilot study has tested the adequacy of measures, has shown that the research protocol is logistically possible, confirms that patient recruitment is not problematic and has informed sample size determination for a substantive trial.

## Appendix 1

### Clinician questionnaire

Instructions

Please place an X, on each line below, depending on how you rate each of the clinical features in relation to the two extremes shown. Measure from the left hand side to the mark. For questions 4–8, please circle as appropriate.

1. Please rate the amount of granulation tissue seen in this patient’s ear.
2. Please rate the amount of erythema seen in this patient’s ear.
3. Please rate the amount of discharge seen in this patient.
4. Is the tympanic membrane intact? (Yes/no)
5. Does the tympanic membrane appear lateralized? (Yes/no)
6. Does the external auditory canal appear to be deformed? (Yes/no)
7. Is there evidence of meatal stenosis? (Yes/no)
8. Please attach a photocopy of the audiogram for this patient.

## Appendix 2

### Patient questionnaire

Instructions

Please indicate, on each line, where each of the below symptoms are in relation to the two extremes shown. Measure from the left hand side to the mark. For question 3, please circle as appropriate.

1. Please rate any ear pain that you may have had since your operation or last outpatient visit.
2. Please rate any discharge that you may have had since your operation or last outpatient visit.
3. Please rate any itching that you may have had since the surgery prior to this appointment.
4. Was a dressing removed from your ear? (Yes/no)
5. If yes please rate any pain that it caused, if any.
